# The Impact of Diabetes Education on Continuous Glucose Monitoring in SUS-Dependent Patients in a Northeastern Brazilian City

**DOI:** 10.3390/life14030320

**Published:** 2024-02-28

**Authors:** Lysandro Pinto Borges, Pamela Chaves de Jesus, Jessiane Bispo de Souza, Deise Maria Rego Rodrigues Silva, Pedro Henrique Macedo Moura, Ronaldy Santana Santos, Marina dos Santos Barreto, Adriana Gibara Guimarães, Lucas Alves da Mota Santana, Dennyson Leandro Mathias da Fonseca, Ikaro Daniel de Carvalho Barreto, Breno de Mello Silva, Carla Raquel Pereira Oliveira, Karla Freire Rezende, Naira Horta Melo, Elenalda Ferreira dos Santos, Carmem Lúcia Matias de Queiroz, Lucia Helena Modesto Xavier, Otávio Cabral-Marques, Eloia Emanuelly Dias Silva

**Affiliations:** 1 Department of Immunology, Institute of Biomedical Sciences, University of São Paulo, São Paulo 05508-220, Brazil; 2 Department of Pharmacy, Health and Biological Sciences Center, Federal University of Sergipe, São Cristóvão 49100-000, Brazil; 3 Graduate Program in Dentistry, Health and Biological Sciences Center, Federal University of Sergipe, Aracaju 49060-102, Brazil; 4 Interunits of Graduate Studies in Bioinformatics, Institute of Mathematics and Statistics (IME), University of São Paulo, São Paulo 05508-220, Brazil; 5 Department of Statistics, Federal University of Pernambuco, Recife 50670-901, Brazil; 6 Department of Biological Science, Federal University of Ouro Preto, Ouro Preto 35400-000, Brazil; 7 Department of Medicine, Health and Biological Sciences Center, Federal University of Sergipe, Aracaju 49060-102, Brazil; 8 Department of Nursing, Health and Biological Sciences Center, Federal University of Sergipe, Aracaju 49060-102, Brazil; 9 Department of Nursing, University of Pernambuco, Recife 50100-010, Brazil; 10 Association of Juvenile Diabetes Brazil (AJD), Água Branca 05003-010, Brazil; 11 Department of Biology, Federal University of Sergipe, São Cristóvão 49100-000, Brazil; 12 Department of Clinical and Toxicological Analyses, School of Pharmaceutical Sciences, University of São Paulo (USP), São Paulo 05508-220, Brazil; 13 Department of Medicine, Division of Molecular Medicine, Laboratory of Medical Investigation 29, University of São Paulo (USP) School of Medicine, São Paulo 05403-010, Brazil; 14 Network of Immunity in Infection, Malignancy, and Autoimmunity (NIIMA), Universal Scientific Education and Research Network (USERN), São Paulo 05508-000, Brazil; 15 Instituto D’Or de Ensino e Pesquisa, São Paulo 01401-002, Brazil

**Keywords:** diabetes, SUS, continuous glucose monitoring system

## Abstract

Background: Diabetes Mellitus (DM) is an important chronic disease that occurs worldwide. Aims: This study aims to investigate how the use of the FreeStyle^®^ Libre system in Unified Health System (SUS) patients impacts diabetes parameters in patients who receive education on proper insulin administration and the use of the continuous monitoring device, as well as how this affects patients without any concomitant multidisciplinary support in Sergipe, Brazil. Methods: We conducted a prospective randomized study in a diabetes clinic in Sergipe, Brazil, using the flash method FreeStyle^®^ Libre (Abbott). The participants were divided into two groups: one receiving diabetes education on CGM (continuous glucose monitoring), while the other did not. Before the intervention, the patient’s treatment motivation and quality of life were assessed using a questionnaire, and baseline levels of glycated hemoglobin were measured using high-performance liquid chromatography (HPLC) and the point of care Alere^TM^ Afinion with boronate fixation. We compared first- and second-phase data with respect to glycated hemoglobin, mean interstitial blood glucose, time on and above target for hypoglycemic and hyperglycemic events, and mean hypoglycemic duration. Results: In group A, which received the diabetes education intervention, there was a significant reduction in average HbA1c levels from 8.6% to 7.9% after 3 months (*p* = 0.001). However, there was no significant difference in average glycemic values. Time above target decreased significantly from 50.62% to 29.43% (*p* = 0.0001), while time below target decreased from 22.90% to 20.21% (*p* = 0.002). There was no significant change in the number of hypoglycemic events, but the duration of hypoglycemia decreased significantly from 130.35 min to 121.18 min after 3 months (*p* = 0.0001). In Group B, there was no significant difference in mean HbA1c levels before (7.07%) and after (7.28%) sensor installation. This group maintained lower HbA1c levels compared to the other group. Average blood glucose levels also remained similar before (148.37 mg/dL) and after (154.65 mg/dL) the intervention. Although the time above the target glucose level increased significantly from 35.94% to 48.17%, the time at target decreased from 50.40% to 37.97%. No significant changes were observed in the time below target, the number of hypoglycemic events, or the duration of hypoglycemia. Conclusions: Our findings indicate that utilizing continuous glucose monitoring technology can enhance glycemic control, particularly in motivated, educated, low-income patients dependent on the SUS. To achieve positive results with FreeStyle Libre, it is imperative to allocate resources for multidisciplinary support.

## 1. Introduction

Diabetes Mellitus (DM) is an important chronic disease that occurs worldwide. Global data suggest that one in six adults in the world has diabetes, which equates to 536.6 million people living with diabetes and an estimated increase of 783.2 million cases of diabetes worldwide by 2045 [1]. The Brazilian population is estimated to be 212.7 million, and the country has the fifth highest incidence of diabetes in the world, behind only China, India, the United States, and Pakistan [2]. Brazil has 16.8 million people with diabetes aged between 20 and 79 years old [2]. The vast majority of these patients depend exclusively on the Unified Health System (SUS), Brazil’s free public health system, to pay for all their treatment, exams, supplies, and consultations [3]. According to data from the IBGE (the Brazilian Institute of Geography and Statistics), 150 million Brazilians depend exclusively on SUS for health care, which means that 7 out of 10 patients would be without care if this system did not exist [4].

It is known that most people with diabetes in Brazil do not maintain their glycated hemoglobin levels below 7%, as recommended by national and international medical societies [5]. This lack of control leads, in the long term, to the development of cardiovascular, renal, ophthalmological, micro and macrovascular, neurological, and metabolic diseases [6]. The effective control of diabetes involves a complex range of factors, such as motivated and up-to-date multidisciplinary health teams, the availability of appointments with endocrinologists, good quality supplies for glycemic monitoring, good quality drugs, therapeutic adherence, dietary control, physical exercises, psychological care, and lifestyle changes.

In Sergipe, the smallest state in Brazil, located in the northeast of the country, there are 94,169 patients with diabetes from a population of 2.22 million people [6]. The Medical Specialty Center of Aracaju (CEMAR) serves around 50,000 patients with diabetes in the municipality, providing all inputs, consultations, and assistance to patients with diabetes via SUS. Most of these patients have some type of comorbidity arising from poor diabetes control, such as diabetic foot, nephropathy, or diabetic retinopathy.

Since 2011, the northeast region has consistently reported the highest incidence of lower limb amputations. In 2019, the states of Alagoas and Sergipe recorded 27 amputations per 100,000 inhabitants [7]. Turning attention to the health costs associated with diabetes, our country allocates approximately 401 million BRL (81 million USD) to cover expenses for free hemodialysis and peritoneal dialysis under the Unified Health System [7]. Notably, Sergipe alone has 1500 kidney patients with diabetes currently undergoing hemodialysis [8].

Adding to the complexities, diabetic retinopathy emerges as a prominent complication of Diabetes Mellitus (DM), standing as the primary cause of blindness among individuals aged 20 to 74. It contributes to approximately 12% of new cases involving reduced visual acuity, hindering individuals from performing their work activities effectively [9].

After having the disease for 20 years, it is estimated that 90% of patients with DM1 and 60% of patients with DM2 have some degree of it. When untreated, there is an estimated rate of evolution to severe blindness of 50% over five years [10]. It is estimated that a 1% decrease in HbA1c values is related to a 35% and 39% reduction in the risk of developing or progression in retinopathy, respectively. Moreover, adequate blood pressure control may be responsible for a reduction in the risk of disease progression by 47% [11]. Adherence to DM treatment can prevent these complications, but an important point is how the patient’s mental health can affect their adherence and predispose them to complications.

Motivation is an aspect of mental health that can influence decision-making and compliance with medical guidelines for the main chronic diseases, including DM [12]. Patient motivation is also an important factor in adherence to the treatment [12]. However, the routine of measurements, applications, or the diet itself can be influenced when the patient is less willing or motivated to continue with the treatment [13]. In this case, there is a possibility that the motivation of patients living with DM may influence glycemic indices throughout treatment [13]. But more than that, the way in which after-care guidance is given can also promote motivation and better adherence to DM treatment [14].

It is well known that controlling glycemic parameters is essential for patients living with diabetes [15]. Among the various practices that can control these parameters, diabetes education is a health education approach focused on the acceptance and continuity of treatment [16]. Diabetes education is focused on dietary re-education, the application or use of insulin in its different routes of administration, in addition to measuring blood glucose and understanding other glycemic parameters [17]. This approach to health is guaranteed to be effective by the guidance given to patients on the protocols and the periodicity and proper use of the devices used to measure blood glucose levels.

Continuous glucose monitoring systems, a type of device for measuring glucose and other glycemic indices, are devices that indicate the mean amount of glucose present in the interstitial fluid [18]. This device consists of a sensor and a reader, which, when approached, can dose the mean glucose and indicate parameters such as the time above target, below target, and at the glycemic target [18]. The indexes measured in real time are presented to the users of this device, who can also be checked by other specialists [18]. In Brazil, this device can only be made available by the Unified Health System through a judicialization process [19]. This process can limit access to the device, excluding other possibilities for better adherence to treatment.

Faced with the finding that uncompensated diabetes leads to the development of comorbidities in the medium and long term and that the associated costs to the SUS will be greater than the costs of prevention through the use of continuous monitoring systems, we investigated how the use of the FreeStyle^®^ Libre system in SUS patients impacts diabetes parameters in patients who receive education in the proper administration of insulin and the use of a continuous monitoring device and patients, as well as the impact on patients without any concomitant multidisciplinary support in Sergipe, Brazil.

## 2. Materials and Methods

**Figure 1:**
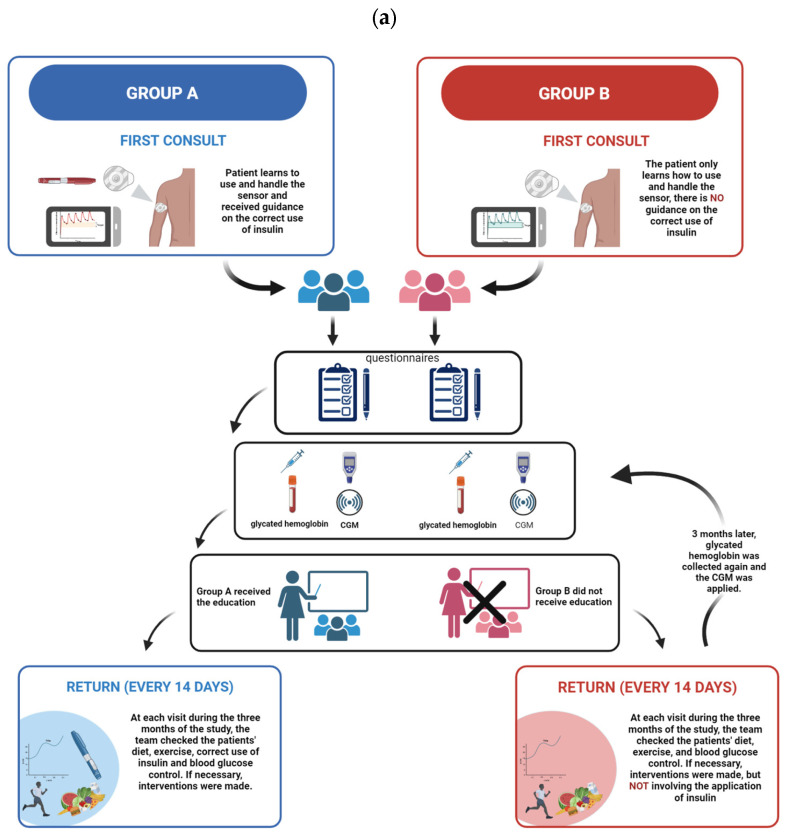

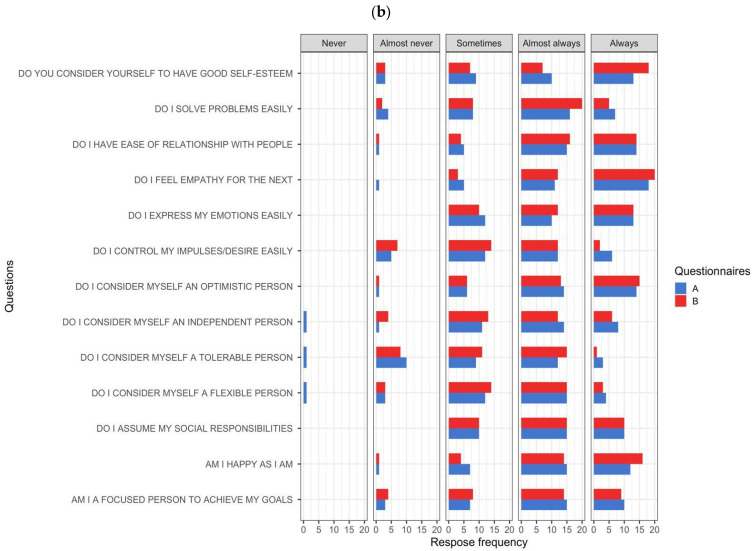
(**a**) Representation of the groups studied in the research: group A (blue) and group B (red) and the organization of the study; (**b**) Frequency of responses from group A (blue) and group B (red) participants. On the left are the questions proposed by the questionnaire. Above, “Never”, “Almost never”, “Sometimes”, “Almost always” and “Always” represent the different levels of agreement and disagreement for the question.

The study was approved by the National Bioethics Committee of the Federal University of Sergipe under the number 3446738. The entire study design is illustrated in Figure 1. We conducted a prospective study that took place in 2019 at the CEMAR diabetic outpatient clinic in Aracaju, Sergipe, in the medical specialties center and was carried out with patients diagnosed with type 1 diabetes because they use insulin as a continuous treatment.

We organized 2 groups of participants, with 35 components each, for the research: 1 that received diabetes education on continuous glucose monitoring and another that did not receive diabetes education on the continuous glucose monitoring device. The study sampling was by convenience, and the eligibility criteria included patients with type 1 diabetes diagnosed more than 2 years ago, aged between 18 and 60 years.

According to the Brazilian Diabetes Society (SBD) [20], the treatment goals for DM1 and DM2 patients consist of an HbA1c below 7.0%. Fasting or preprandial blood glucose should be between 80 and 130 mg/dL. For 2 h postprandial blood glucose, a value below 180 mg/dL is recommended. These values should be between 90 and 150 mg/dL at bedtime.

Exclusion criteria were patients under the age of 18 and over the age of 60, with diabetes diagnosed less than 2 years ago, due to the occurrence of greater glycemic instability and adequacy to treatment and considering the greater understanding of the use of and adherence to the monitoring system. Additionally, an exclusion criterion was failure to monitor blood glucose 6 times a day due to the need to capture patients engaged in blood glucose monitoring. We also excluded patients suffering from kidney failure, retinopathy, liver disease, and chronic comorbidities because the medical needs of these pathologies require intensive care that could jeopardize the study’s follow-up routine. In addition to our aforementioned exclusion criteria, patients who did not attend the fortnightly follow-up, a schedule of appointments that took place every 15 days during the research period in order to change and adjust the continuous glucose monitoring device; lost the sensor; had problems with the sensor; or did not take the sensor reading more than six times a day were excluded from the study due to the loss of continuity of the follow-up by the researchers. Based on all these exclusion criteria, we arrived at a total of 70 patients. After being included according to our study’s inclusion and exclusion criteria, the patients were randomly assigned to the groups.

### 2.1. Diabetes Education

In the first consultation with the doctor and the pharmacist, the continuous monitoring system (FreeStyle^®^ Libre, Abbott) was installed, and the patient was given information on the system and how to use it. One group, which we call group A, with 35 participants, received diabetes education focusing on insulin therapy and the correct use of the continuous glucose monitoring device. Group B, which included 35 participants, received the device but no education about diabetes or the correct administration of insulin by our team; however, we cannot say that these patients applied it correctly or already received adequate guidance during their treatment. Although the patients who approved participation in the research did not receive diabetes education on insulin therapy, they were given guidance on the use of the device’s sensors and readers, as well as the importance of using the device. They were also informed of the availability of the technical team to help them and the need to exchange the device with our team.

Group A received guidance on the appropriate temperature for storing and homogenizing the insulin pen, on the different sites on the body that can receive insulin administration, and on the importance of coating to avoid tissue insulin resistance resulting from lipohypertrophy. They were also instructed in dietary reeducation to prevent hypoglycemia and were advised to consume foods or liquids with rapid glycemic absorption. All the instructions were based on the guidelines of the Brazilian Diabetes Society to promote diabetes education for patients living with the disease.

In contrast, group B received guidance on the use of the continuous glucose monitoring device, the handling of the reader, and the different times of day to check the monitoring, avoiding measurement immediately after meals, avoiding conflict with glycemic peaks, and carrying out a more accurate measurement of interstitial glucose.

All the patients completed a Summary of Diabetes Self-Care Activities questionnaire adapted and validated by Michels et al. (2010) to assess their motivation and self-esteem with respect to the treatment of diabetes [21]. The guidance given during consultations was based on the guidelines of the Brazilian Diabetes Society [https://diretriz.diabetes.org.br/ (accessed on 3 June 2023)] [20] and instructions provided by the device manufacturer’s website [https://www.freestyle.abbott/pt-pt/tutoriais.html (accessed on 3 June 2023)] [19].

### 2.2. Collection and Analysis of HbA1C

To analyze glycated hemoglobin, the study used two methodologies: the Alere Afinion™ AS100 Analyzer (Alere Technologies AS, Oslo, Norway), a point-of-care analyzer that provides quantitative results within 3 min to check the level of HbA1C, and high-performance liquid chromatography (HPLC), which identifies hemoglobin A1c by separating and quantifying hemoglobin components.

To measure the HbA1c with the Afinion™, we collect 1.5 μL of capillary blood samples from patients who will be checking their sensor glucose and analyze HbA1C with the Afinion™ HbA1C Test Kit (Abbott, Oslo, Norway). The collected sample was inserted into the test to measure glycated hemoglobin. The results displayed by the device were recorded and tabulated.

To measure glycated hemoglobin using the HPLC method, we collected 4 mL of venous blood in an EDTA tube to preserve the red blood cells and prevent the sample from clotting. The measurement of glycated hemoglobin by high-performance liquid chromatography is the reference method for assessing glycemic oscillation. The collected sample was processed on the BioRad™ D10 analyzer. The results presented by the two HbA1c measurement methods were compared using a mean calculation and found to be similar. Afterward, the analyzer presented results that were given to the patients and reserved for use in the study.

The results presented by the two HbA1c measurement methods were compared and found to be similar. Subsequently, the analyzer presented results that were given to the patients and reserved for use in the study.

Every 14 days, the patients returned to change the glucose sensor and check their general condition. All clinical information (time below target, time above target, and time on target, as well as mean glycemia and duration of hyperglycemic and hypoglycemic events) was collected from each patient’s monitoring system. At three months, the patients had their last consultation, the data collected were then tabulated in the software Microsoft^®^ Excel 365, and the statistical analysis was performed.

### 2.3. Statistical Analysis

The statistical data analysis was expressed as frequencies and percentages. Continuous variables were expressed as the mean, with a 95% confidence interval and standard deviation. A paired *t*-test was performed to determine the significant difference between the means. The False Discovery Rate (FDR) was also checked for better control of the results. Statistical analyses were performed using IBM^®^ SPSS^®^ Statistics software (version 26.0 for Windows).

We also calculated the sample size for the main study scenario of a two-sided paired *t*-test, 5% significant difference level, medium size effect (Cohen’s d = 0.5), and 80% power, and we achieved 35 participants per group, totaling 70.

## 3. Results

A total of 70 patients with type 1 diabetes were surveyed, and all participants provided informed consent. Group A had thirteen (37.14%) male participants and twenty-two (62.86%) female participants. In the group B patients, eighteen (52.94%) were male and seventeen (47.06%) were female.

With regard to age coverage, group A contained 9 (25.71%) participants aged between 20 and 30, 15 (42.85%) patients aged between 30 and 50, and 11 (31.42%) patients aged between 50 and 60. Group B contained 7 (20.01%) participants aged between 20 and 30, 9 (25.71%) patients aged between 30 and 50, and 19 (54.28%) patients aged between 50 and 60.

### 3.1. Answers to the Questionnaire

Overall, for the 13 questions, we obtained 455 specific answers from each group. Group A obtained an overall frequency of 3 “Never” answers, characterizing 0.65% of the total answers, while “Almost never” appeared 33 (7.25%) times as an answer to the questions. The answer “Sometimes” obtained an overall frequency of 113 (24.83%), while “Almost always” obtained a higher frequency, with 174 answers, making up 38.24% of the answers in the questionnaire, and “Always” accounted for 132 answers, making up 24.83% of the total answers in the questionnaire. On the other hand, Group B did not select “Never” as an answer to any of the questions. “Almost never” was ticked on 34 occasions (7.47%). “Sometimes” was chosen with a total frequency of 112 answers (24.71%). Meanwhile, “Almost always” was the most frequent answer, appearing 177 times, which represents 38.90% of the answers in the questionnaire. Finally, “Always” was selected in 132 answers, amounting to 29.01% of the total set of answers in the questionnaire. The results of the questionnaire for both groups can be seen in Figure 1b, as well as in Supplementary Materials.

### 3.2. Group A Patients

**Figure 2:**
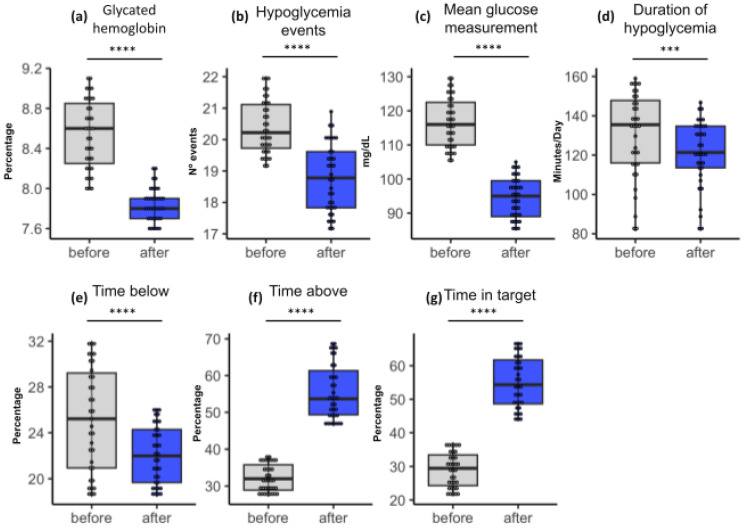
Group A boxplots showing statistical results among paired samples. The blue color in the box represents the values observed in the last check. The upper and lower limits of the boxes, in blue, indicate the result range. The horizontal line inside each box indicates the median. The vertical lines indicate the outliers. The asterisks indicate significance (*** = 0.01; **** = 0.001). The significance presented refers not only to the *p*-value but also to the False Discovery Rate (FDR) in the pairing.

About the quantitative data from the analysis of glycated hemoglobin (Figure 2a), the patients who received diabetes education showed a reduction in their mean HbA1c when compared to the mean of the first visit. In the paired T-test, the mean glycated hemoglobin before the installation of the sensor with the educational intervention was 8.6% with a confidence interval (95%CI) between 8.1 and 9.0 and a standard deviation (SD) of 1.3, and after 3 months, it was 7.9% (95%CI = 7.6–8.1; SD 0.9) with a *p*-value = 0.001.

Figure 2 shows the clinical differences between patients of groups A and B before follow-up with the FreeStyle^®^ Libre system and after follow-up. It is also possible to analyze the presence of outliers in all parameters, which are the points outside the box diagram that are furthest from the median values. Most of the results were below or above the median, as in the case of glycated hemoglobin, where no statistical differences could be seen before and after the use of FreeStyle^®^ Libre; some patients had levels below the median (<6.6%) before using the technology, and this changed soon after its use, with some being above and others below the median.

The glycemic mean (Figure 2c) of these patients decreased from a previous mean of 116.57 mg/dL (95%CI = 114.14–119.00; SD 7.36) to a mean of 94.69 mg/dL (95%CI = 92.70–96.68; SD 6.01) with a *p*-value of 0.0983. The analysis indicated that there was no significant difference between the previous and subsequent glycemic values.

The mean time above the target (Figure 2f) indicated by the sensor rose to 34.62% (95%CI = 27.05–42.19; SD 7.57), and after 3 months, this rose to 57.43% (95%CI = 53.74–61.12; SD 3.69). This indicates a significant difference between the time above the target values presented before and after the intervention (*p* = 0.0001).

At the glycemic target (Figure 2g), the initial value was 28.48% (95%CI = 23.28–33.68; SD 5.20), and after 3 months, it was 55.36% (95%CI = 47.76–62.96; SD 7.60) with a *p*-value = 0.111. Statistical analysis showed that there was no significant difference between the values compared.

The mean time below the target (Figure 2e) was 25.90% (95%CI = 21.31–30.49; SD 4.59), and after 3 months, this was 20.21% (95%CI = 17.64–22.78; SD 2.57), with a significant decrease (*p* = 0.002).

The mean number of hypoglycemic events (Figure 2b) at the first consultation was 20.42 (95%CI = 20.14–20.70; SD 0.87), and after 3 months, it was 18.78 (95%CI = 18.43–19.13; SD 1.08) with a *p*-value = 0.650. The values for hypoglycemia events before and after the intervention were not significant. Finally, the mean duration of hypoglycemia (Figure 2d) was 130.35 min at the first visit (95%CI = 123.10–137.61; SD 21.90), and after 3 months, it was 121.18 min (95%CI = 115.53–126.83; SD 17.07) with a *p*-value = 0.0001.

### 3.3. Group B Patients

**Figure 3:**
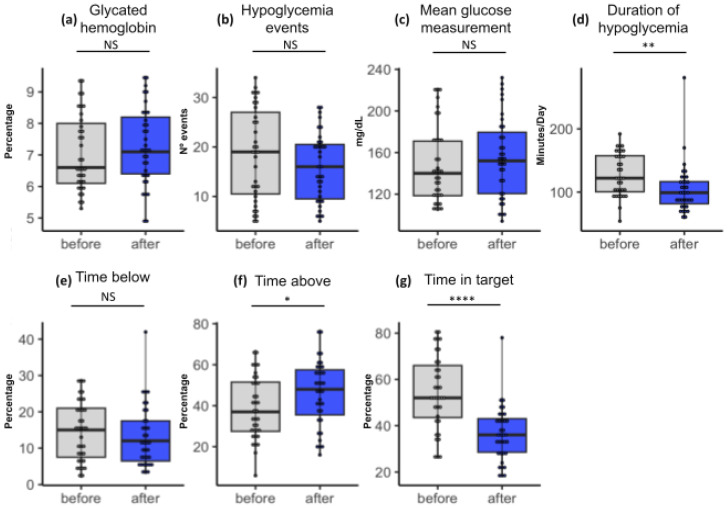
Group B boxplots showing statistical results among paired samples. The blue color in the box represents the values observed in the last check. The upper and lower limits of the boxes, in blue, indicate the result range. The horizontal line inside each box indicates the median. The vertical lines indicate the outliers. The asterisks indicate significance (* = 0.1; ** = 0.01; **** = 0.001). The significance presented refers not only to the *p*-value but also to the False Discovery Rate (FDR) in the pairing. NS: not significant.

The mean glycated hemoglobin (Figure 3a) before sensor installation was 7.07% (95%CI = 6.67–7.46; SD 1.20). Three months after sensor installation, the mean HbA1C was 7.28% (95%CI = 6.87–7.68; SD 1.22). We observed that there was no statistical difference before and after the monitoring (*p* = 0.19). In addition, it can be seen that this group had a lower HbA1c than group A. Therefore, despite showing an increase in the mean after three months of study, this group is within the values recommended by the literature (equal to or below 7.5%).

The mean glycemia (Figure 3c) before and after the intervention were 148.37 mg/dL (95%CI = 136.05–160.69; SD 37.2) and 154.65 (95%CI = 141.77–167.53; SD 38.9), respectively. The statistical analysis indicated that there were no significant differences between monitoring (*p* = 0.084).

On the other hand, the mean time above the glycemic target (Figure 3f) indicated was 38.94% (95%CI = 23.78–54.10; SD 15.26), and after 3 months, this rose to 48.17% (95%CI = 31.99–64.35; SD 16.18), and there was a statistically significant difference between initial values and values at 13 weeks (*p* = 0.007).

Regarding the mean time of the glycemic target (Figure 3g), the initial value was 50.40% (95%CI = 34.22–66.58; SD 16.00), and after 3 months, it was 37.97% (95%CI = 25.04–50.90; SD 12.93). There was a statistically significant difference between initial values and values at 13 weeks (*p* = 0.0001).

Furthermore, the mean time below the target (Figure 3e) was 17.66% (95%CI = 9.56–25.76; SD 8.1), and after 3 months, this was 13.86% (95%CI = 3.96–23.76; SD 9.9). There was no statistically significant difference between the two times (*p* = 0.320).

The mean number of hypoglycemic events (Figure 3b) at the first consultation was 18.54 (95%CI = 15.46–21.62; SD 9.30), and after 3 months, it was 15.88 (95%CI = 13.59–18.17; SD 6.93). There was no statistically significant difference between the two times (*p* = 0.093).

Finally, the mean duration of hypoglycemia (Figure 3d) was 128.08 min at the first visit (95%CI = 116.98–139.18; SD 33.52), and after 3 months, it was 104.42 min (95%CI = 91.26–117.58; SD 39.73). There was no statistically significant difference between times (*p* = 0.058).

In summary, the analyzed data suggest that there were only two negative changes in parameters (time above target and time on target), and the other parameters analyzed did not show any statistically significant differences between the first consultation and after three months of use of the continuous monitoring system in Figure 3.

## 4. Discussion

In this study, we monitored patients with DM1 who received the continuous glucose monitoring sensor for three months. The patients were separated into two groups (groups A and B) so that we could assess the effect of using the system on patients who did and did not receive the education focused on the correct use of insulin carried out by our team.

We noticed that group A had more female participants, while group B showed a balance between the sexes. These sociodemographic results regarding participants’ sex reflect the population context of the state where the study was conducted. It turns out that the state of Sergipe has a population of 2.376.447, where 48.3% of it is composed of males and 51.6% is composed of females [22]. Thus, our study may have been influenced by this perspective of population distribution. In addition, the glycemic control results of the groups may be affected by the gender of the participants, since studies show that women tend to show more proactive behavior in monitoring their signs and symptoms compared to men [23,24]. This disparity in behavior can have a significant influence on the management and results of Diabetes Mellitus treatment.

Regarding the age group results, we observed disparities between the groups, particularly in the age ranges of 30 to 50 years and 50 to 60 years. Group A had more participants aged between 30 and 50 years, while group B had the majority of participants aged between 50 and 60 years. In this case, especially in group B, this disparity may influence glycemic control and the use of CGM itself, as patients in this age range may experience a more complex perspective in managing DM, either due to the presence of other associated comorbidities or even due to dependence on care and greater fragility in dealing with the self-care routine, applications, and measurements [25].

With the results identified, we could see that both groups were motivated. For the most part, we had responses that indicated a routine of good self-esteem and motivation in the patients. When analyzing the answers to the questionnaire on self-esteem and emotional well-being, we observed a variety of frequencies for each question. The answers “Almost always” and “Sometimes” were the most common in several questions, suggesting that most participants experience a combination of positive and some negative feelings towards themselves while living with Diabetes Mellitus.

Regarding the results of the glycated hemoglobin, group A showed a reduction in HbA1c values, indicating that the educational intervention for insulin therapy in diabetes is effective for efficient glycemic control. In addition, multi-professional monitoring also stands out in terms of controlling patients’ blood glucose levels and improving lifestyle habits related to diabetes control. However, the glycated hemoglobin levels of group B did not change in a statistically significant way during the three months of evaluation, indicating that the use of the flash system alone, without other complementary interventions, cannot reduce glycated hemoglobin levels in the population studied [14,26,27,28,29,30,31,32,33].

We suggest that it is essential for patients to be monitored by a qualified and motivated multidisciplinary team in order to achieve a possible reduction in HbA1C and the importance of maintaining target glycemic levels (70 to 180 mg/dL) to avoid the comorbidities resulting from diabetes. This is achieved by different professional visions in their respective fields with regard to correct guidance on changes in lifestyle habits, exercise, diet, and the correct use of medication. However, we should point out that the short follow-up period (3 months) applied in this study may have an impact on these glycemic change values. With regard to mean glycemic levels, several studies suggested that the use of the flash system can help produce a reduction in levels [14,33]. Other studies reported similar results to ours with the use of continuous monitoring systems that do not affect mean glycemia [34].

In the context of FreeStyle Libre, “glycemic target” refers to the goal of blood glucose control, in this case, the level of glucose you want to maintain within a specific range. The glycemic target can vary from person to person, depending on various factors such as age, type of diabetes, medical history, and lifestyle. Regarding the time above target values, group A showed a significant decrease, indicating a reduction in the time spent in hyperglycemia. The increase in the time above the target that we found in the group B patients after 13 weeks is associated with increased hyperglycemia since these patients spent more time with blood glucose above the therapeutic target recommended by the SBD guidelines. Studies suggest that the use of the flash system can induce a false sense of glycemic control on the part of the patient, causing them to decrease their concern about blood glucose levels, negatively affecting behavior and resulting in increased glucose levels [35,36,37,38,39]. On the other hand, other studies reported a reduction in hyperglycemia after the use of the flash system in patients assisted by a multidisciplinary team [40,41,42,43,44,45,46,47,48,49].

Regarding time in target, this is an important marker in diabetes control [50], with the various medical societies in Brazil and around the world considering that time in target values of 70% or above indicate good glycemic control and the reduction of long-term comorbidities if the patient maintains these levels throughout life. The group A time in the target showed a significant increase, indicating that the use of the sensor is efficient for glycemic monitoring as long as diabetes education is carried out efficiently. Contrary to what was expected and observed in the literature, our study observed that group B had a significant decrease in time on target after 3 months of treatment using the FreeStyle^®^ Libre. The most likely hypothesis for this finding is the patient’s lack of knowledge of the importance of the time in target [51], lack of education in diabetes [34], and a false sense of control using the new system, with non-existent nutritional and pharmaceutical follow-up, which leads to increased time on target as observed in the study [27,33].

Hypoglycemia is one of the worst symptoms of the patient with diabetes and can lead to events such as fainting and a feeling of malaise, which, at worst, can result in serious accidents or hypoglycemic coma [51,52,53]. Analyzing the data from our study, we observed that in group A, there was a decrease in hypoglycemia. This indicates that the use of the sensor is effective in reducing hypoglycemia in insulin-dependent patients. However, group B, which did not receive an educational intervention, showed a non-significant decrease in the number of hypoglycemia events.

The duration of hypoglycemia decreased, indicating that the use of the continuous monitoring device makes it easier to monitor hypoglycemia. In addition to this study, several other studies showed that the use of the flash system was associated with a reduction in hypoglycemia [54,55,56]. We hypothesize that patients may be able to reduce hypoglycemia with the use of a technological system, especially when they are aware of how to control it through diabetes education and other forms of support [51,52].

This study has some limitations. Even though the responses to the questionnaire indicated that the patients had good treatment motivation, this may be based on biased positive responses to please the researchers. Our sample size may not have been sufficient to represent the entire population of patients living with diabetes in the state of Sergipe. This limitation was mainly due to the limited availability of continuous monitoring devices and the cost of carrying out the study. More studies with larger sample sizes are required to evaluate the effects of using a continuous monitoring system in SUS patients from low socioeconomic groups.

## 5. Conclusions

Based on our results, we can assume that the use of continuous glucose monitoring technology for glycemic control in low-income patients dependent on the SUS can help improve glycemic indices, especially in motivated patients who received diabetes education. For these systems to be used in a cost-effective manner and produce more positive results, it is necessary to allocate resources to provide multidisciplinary support to patients. This means that, in the long term, properly supported use of these systems will help to reduce the significant and growing costs of treating the comorbidities associated with diabetes. With careful planning, the increase in spending in the short term will result in an even greater reduction in long-term health costs related to diabetes and a significant increase in the health and quality of life of patients served by the SUS.

## Data Availability

If you are interested in further data on the results, please contact the corresponding author.

## Supplementary Materials

The following supporting information can be downloaded at: https://www.mdpi.com/article/10.3390/life14030320/s1, Questionnaire answers and frequency tables.
